# Long-Term Effect of Crop Rotation and Fertilisation on Bioavailability and Fractionation of Copper in Soil on the Loess Plateau in Northwest China

**DOI:** 10.1371/journal.pone.0145370

**Published:** 2015-12-22

**Authors:** Yifei Zang, Xiaorong Wei, Mingde Hao

**Affiliations:** 1 College of Natural Resources and Environment, Northwest A & F University, Yangling, Shaanxi, China; 2 Institute of Soil and Water Conservation, Chinese Academy of Sciences and Ministry of Water Resources, Yangling, Shaanxi, China; Henan Agricultural Univerisity, CHINA

## Abstract

The bioavailability and fractionation of Cu reflect its deliverability in soil. Little research has investigated Cu supply to crops in soil under long-term rotation and fertilisation on the Loess Plateau. A field experiment was conducted in randomized complete block design to determine the bioavailability and distribution of Cu fractions in a Heilu soil (Calcaric Regosol) after 18 years of rotation and fertilisation. The experiment started in 1984, including five cropping systems (fallow control, alfalfa cropping, maize cropping, winter wheat cropping, and grain-legume rotation of pea/winter wheat/winter wheat + millet) and five fertiliser treatments (unfertilised control, N, P, N + P, and N + P + manure). Soil samples were collected in 2002 for chemical analysis. Available Cu was assessed by diethylene triamine pentaacetic acid (DTPA) extraction, and Cu was fractionated by sequential extraction. Results showed that DTPA-Cu was lower in cropping systems compared with fallow control. Application of fertilisers resulted in no remarkable changes in DTPA-Cu compared with unfertilised control. Correlation and path analyses revealed that soil pH and CaCO_3_ directly affected Cu bioavailability, whereas available P indirectly affected Cu bioavailability. The concentrations of Cu fractions (carbonate and Fe/Al oxides) in the plough layer were lower in cropping systems, while the values in the plough sole were higher under grain-legume rotation relative to fallow control. Manure with NP fertiliser increased Cu fractions bound to organic matter and minerals in the plough layer, and its effects in the plough sole varied with cropping systems. The direct sources (organic-matter-bound fraction and carbonate-bound fraction) of available Cu contributed much to Cu bioavailability. The mineral-bound fraction of Cu acted as an indicator of Cu supply potential in the soil.

## Introduction

Copper (Cu) is an essential micronutrient in crop production. The bioavailability of Cu in soil is regulated by its adsorption, desorption and solubility [1, 2]. The adsorption and desorption processes of Cu strongly depend on the soil microenvironment and chemical properties, such as pH, CaCO_3_, organic matter, and available phosphorous (P) levels [2–5]. Moreover, cropping systems and fertilisation practices affect the bioavailability of Cu in soil [6–8]. A 9-year fertilisation study found that manure accelerated the depletion of available Cu in a purple paddy soil in southwest China [9].

Cu can present in different forms and associate with various soil constituents in multiple ways, which determine its bioavailability and mobility in soils [10, 11]. Investigators have approached the problem of Cu in soil by sequential extractions, with a view to characterizing various available forms of Cu [12]. The water-soluble, exchangeable, and organically-complexed Cu forms are available to plants, whereas Cu occluded in oxides of iron (Fe), aluminium (Al) and manganese (Mn) as well as primary and secondary minerals are not readily available [13, 14].

Owing to the high carbonate, high pH and low total Cu levels, Cu deficiency often occurs in soils on the Loess Plateau of China [15]. In this region, soil available Cu, extracted with DTPA, ranges from 0.01 to 4.2 mg kg^−1^, with an average concentration of 0.93 mg kg^−1^ [16]. More than 20% of the loess soils have been found to contain available Cu less than the critical level (0.5 mg kg^−1^) for crop nutrient deficiency in this area [16].

In recent decades, the use of high-yield crop varieties with high nutrient demands has expanded sharply, making the problem of Cu deficiency increasingly evident [15]. Meanwhile, there are substantial improvements in crop yield and increases in multiple cropping index, with increased application of major fertilisers. As a consequence, soil nutrients become imbalanced in agricultural fields wherein the effect of micronutrients appears more evident for improving crop yield. Yu and Peng had founded the yield increasing effect of soil micronutrients, but it differed from soil type on the Loess Plateau [16]. Therefore, micronutrient fertilisers should be applied according to soil type in different regions.

Thus far, little research has investigated the bioavailability and fractionation of Cu in soil under crop rotations and fertilisation on the Loess Plateau. Thus, it remains difficult to quantitatively evaluate soil Cu concentration and appropriately use micronutrient fertilisers for different crops. Long-term field experimental plots under varying cropping systems and fertilisation practices provide an opportunity to examine the effects of management history on the bioavailability and fractionation of micronutrients in soil.

In this study, an 18-year experiment was conducted to examine the effect of different cropping systems and fertilisation practices on the bioavailability and fractionation of Cu in soil in the southern part of the Loess Plateau.

## Materials and Methods

### Experimental site and soil characterization

The field experiment started in September 1984 at the Agro-ecological Experimental Station (35°12′N, 107°40′E) affiliated to the Chinese Academy of Sciences. The study site is located in Changwu County of Shaanxi Province, as part of the southern Loess Plateau, China. The average altitude is 1200 m above sea level, the average annual temperature is 9.2 ± 2.3°C, and the average annual precipitation is 578 ± 69 mm. The soil is a Heilu soil in the Chinese Soil Taxonomic System, and a Calcarid Regosol according to the FAO/UNESCO Classification System [17]. Prior to the start of the experiment, the surface soil (0–20 cm) had a pH of 8.1, and contained 10.5 g organic matter kg^−1^, 37.0 mg available nitrogen kg^−1^, 3.0 mg available phosphorous kg ^−1^, and 1.1 mg DTPA extractable Cu kg ^−1^ [18].

### Experiment design and management

The experiment was conducted in a randomized complete block design with a split-plot arrangement. Cropping system and fertiliser were the main plot and sub-plot treatments, respectively. Five main plot treatments included: fallow control (FW), continuous cropping of alfalfa (AC, *Medicago sativa* L.), continuous cropping of maize (MC, *Zea mays* L.), continuous cropping of winter wheat (WC, *Triticum aestivum* L.), and 1-year grain-legume rotation (GLR) of pea (*Pisum sativum* L.)/winter wheat/winter wheat + millet (*Panicum miliaceum* L.). Five sub-plot treatments included: unfertilised control (Ctrl), nitrogen (N), P, N + P (NP), and N + P + manure (NPM). According to local fertilisation practice and N-fixing ability in crops, the fertiliser treatments varied in different cropping systems: AC received Ctrl, P, and NPM; MC received NP and NPM; WC received Ctrl, N, P, and NPM; and GLR received Ctrl, P, NP, and NPM. The FW, AC, MC, WC and GLR had one, three, two, four, and four treatments, respectively. All the 14 treatments were replicated three times, and a total of 42 plots were included in the experiment. Each plot was 10.3 m × 6.5 m.

N and P were applied in the forms of urea (120 kg N ha^-1^) and superphosphate (26 kg P ha^-1^), respectively. Manure (75 t ha^-1^) came from cattle with total N content of 1.97 g kg^-1^, available N of 91 mg kg^-1^, total P of 0.97 g kg^-1^, and available P of 115 mg kg^-1^. Cu content was 9 mg kg^-1^ for P fertiliser and 19 mg kg^-1^ for manure.

Crop cultivars, seeding rates, and sowing and harvesting periods are shown in our previous work [18]. Crop management used was routine practices in the study region. Prior to seeding, fertilisers were surface-applied, and the soil was then ploughed twice by indigenous ploughing (cattle-drawn moldboard country plough) to a depth of ~20 cm. Maize was sown in rows 60 cm apart, wheat and pea were sown in rows 25 cm apart, and clover and millet were broadcasted. After sowing, the seeds were covered by raking the soil.

Legume crops were harvested twice each year for hay. Mature grain crops were harvested at ground level. After the straw and grain were removed, the soil was ploughed twice to a depth of ~20 cm. The FW plots were ploughed twice in June and twice in September each year, without cropping or fertilisation. In all treatments, weeds were removed by hand.

### Soil sampling and laboratory analysis

Soil sampling was conducted in 2002. Five random cores were collected from the plough layer (0–20 cm) and plough sole (20–40 cm) in each plot with a tube auger 5 cm in diameter. Large pieces of stubble and root were removed by hand. Soil samples were air-dried and mechanically ground to pass through 1- and 0.25-mm nylon sieves before laboratory analysis.

Soil pH was determined in a suspension (soil: water = 1: 2, W/W) with a calibrated PHBJ-260 glass electrode (Leici, Shanghai, China). Organic matter was measured by the titration method [19]. Available P was determined by the Olsen method [20]. CaCO_3_ was tested by manometry [21].

Available Cu was obtained from 10 g of soil (≤1 mm) with 20 mL of extraction solution containing 0.005 mol L^-1^ DTPA, 0.1 mol·L^-1^ triethanolamine, and 0.01 mol L^-1^ CaCl_2_ (pH 7.30) [22]. After 2 h of continuous shaking at 25°C, the soil suspension was centrifuged and then filtered through Whatman Grade 5 qualitative filter paper. The resulting supernatant was stored in a polyethylene bottle at 4°C before Cu analysis.

Cu was fractionated using a modified sequential extraction scheme according to the work of Tessier et al. [23] and Shuman [24]. Five gram of sieved soil (≤0.25 mm) was extracted in 80 mL polypropylene centrifuge tubes to minimize the loss of solid materials. Each of the chemical fractions was handled as described in detail previously [25].

Step (1): The exchangeable fraction (Ex-Cu) was extracted from soil samples with 25 mL of 1 mol·L^-1^ NH_4_NO_3_ (pH 7.0) on a shaker (25°C, 2 h).
Step (2): The carbonate-bound fraction (Carb-Cu) was extracted from the residue of step (1) with 25 mL of 1 mol·L^-1^ NaOAc (pH 5.0) on a shaker (25°C, 5 h).
Step (3): The Fe or Mn oxide-bound fraction (Ox-Cu) was extracted from the residue of step (2) with 55 mL of 0.04 mol L^-1^ NH_2_OHHCl in HOAc (25%, V/V) in a water bath with occasional agitation (95 ± 3°C, 5 h).
Step (4): The organic-matter-bound fraction (Om-Cu) was extracted from the residue of step (3) with 3 mL of 0.02 mol L^-1^ HNO_3_ and 5 mL of 30% H_2_O_2_ (pH 2.0) in a water bath with intermittent agitation (85 ± 2°C, 2 h), followed by an additional extraction with 5 mL of 30% H_2_O_2_ (pH 2.0) under the same conditions (85 ± 2°C, 3 h). The residue was cooled and then extracted with 50 mL of 1 mol L^-1^ NH_4_NO_3_ (pH 7.0) on a shaker (25°C, 2 h).
Step (5): The mineral-bound fraction (Min-Cu) was extracted from the residue of step (4) by heating (180°C) and subsequent digestion with a mixture of HNO_3_–HClO_4_–HF (200°C).

After each successive extraction, the extract was centrifuged at 4000 rpm for 10 min and passed through Grade 5 filter paper. The supernatant was collected and stored in a polyethylene bottle at 4°C. The residue was washed with deionized water and then use in the next extraction step. Cu in the extract was analysed using a SpectrAA-220 Zeeman atomic absorption spectrometer (Varian, Palo Alto, CA, USA). All chemicals used were of analytical grade.

### Statistical analysis

Data are mean values ± standard error. Variance, correlation, path and factor analyses were conducted using SAS (SAS Institute Inc., Cary, NC, USA) [26]. Path analysis is a statistical technique that partitions correlations into direct and indirect effects, and attempts to differentiate between correlation and causation. This technique also features multiple linear regressions and generates standardized partial regression coefficients (path coefficients) [27]. The factor analysis technique, which allows a considerable reduction in the number of variables and the detection of structure in the relationships between variables, has been successfully used for evaluation of heavy metals and their relations with metal fractions by Maizet et al. [28]. All data were subjected to ANOVA, and when significant differences (*P*<0.05) were detected, Duncan’s new multiple rang test was performed to separate means.

## Results

### Distribution of DTPA-Cu in soil

**Fig 1:**
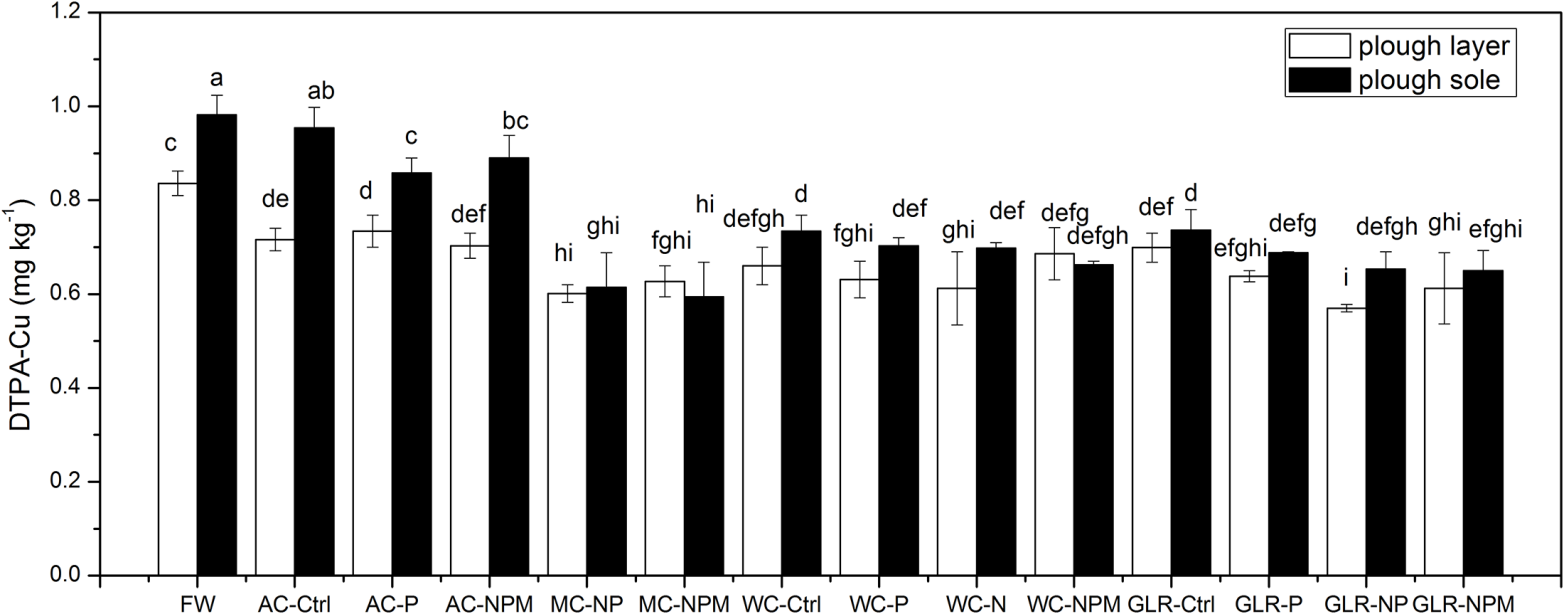
Soil DTPA-Cu concentrations in cropping systems with fertilization treatments. Different letters indicate a significant difference at *p*<0.05. Error bars represent standard errors of the mean (*n = 3*). Cropping systems: FW, fallow control; AC, continuous alfalfa; MC, continuous maize; WC, continuous winter wheat; GLR, grain-legume rotation. Fertiliser treatments: Ctrl, unfertilised control; N, nitrogen; P, phosphorus; NP, N + P; NPM, N + P + manure.

The concentrations of DTPA-Cu ranged from 0.52 to 1.02 mg kg^-1^, with an average concentration of 0.71 mg kg^-1^. A comparison of different systems revealed that DTPA-Cu concentrations were lower in the cropping systems compared with FW (Fig 1). The values of WC-Ctrl for both the plough layer and plough sole were the lowest among the unfertilised treatments, which showed 21.1% and 25.3% decreases compared with FW, respectively. DTPA-Cu in the plough layer was lower in AC-Ctrl than in FW, while no difference occurred in the plough sole.

A comparison of fertilisation treatments within each cropping system indicated that the application of fertilisers had no effect on DTPA-Cu concentration in AC, WC, or MC, and different fertilisers made no differences in the plough layer of these cropping systems (Fig 1). Compared with the unfertilised treatment, the application of NP and NPM decreased DTPA-Cu for GLR.

### Cu fractions in cropping systems

**Fig 2:**
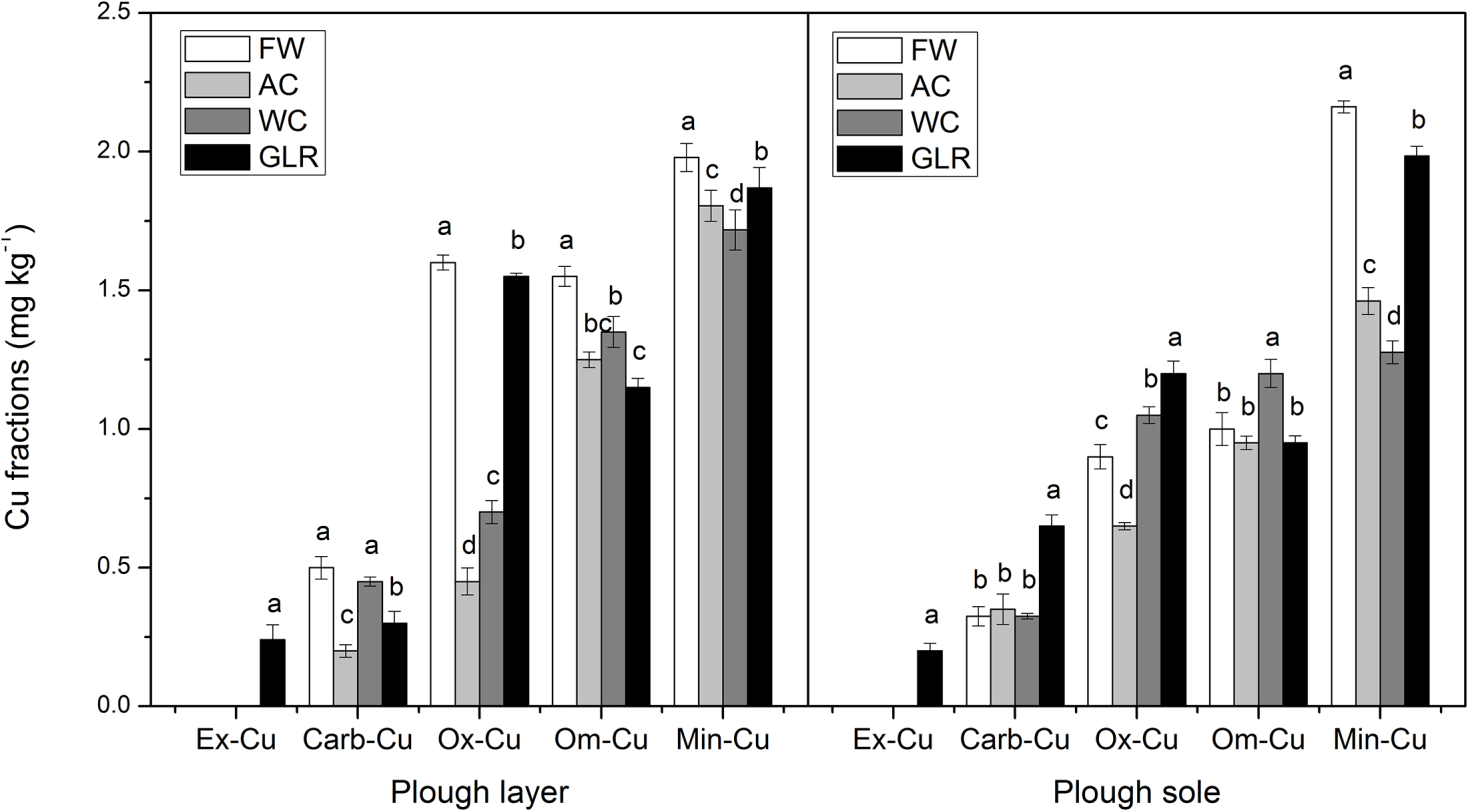
Soil Cu fractions in different cropping systems. The unit for Min-Cu is 10 mg·kg^-1^. Different letters indicate a significant difference at *p*<0.05. Error bars represent standard errors of the mean (*n = 3*). Cropping systems: FW, fallow control; AC, continuous alfalfa; MC, continuous maize; WC, continuous winter wheat; GLR, grain-legume rotation. Cu fractions: Ex-Cu, exchangeable Cu; Carb-Cu, carbonate-bound Cu; Ox-Cu, oxide-bound Cu; Om-Cu, organic-matter-bound Cu; and Min-Cu, mineral-bound Cu.

Total Cu ranged from 15 to 28 mg kg^-1^, which was mainly bound to minerals. Ex-Cu was below the minimum detection limit in most treatments, accounting for at most 1.1% of the total Cu (Fig 2). Carb-Cu was <0.7 mg kg^-1^ and accounted for 1.1–2.9% of the total Cu. Ox-Cu ranged from 0.45 to 1.6 mg kg^-1^, corresponding to 2.3–7.1% of the total Cu. Om-Cu accounted for 4.2–7.8% of the total Cu in soil, behind Min-Cu with a proportion of 83–91%.

Ex-Cu was below the minimum detection limit in FW, AC and WC, while it reached 0.24 mg kg^-1^ in the plough layer and 0.20 mg kg^-1^ in the plough sole of GLR (Fig 2). In terms of Carb-Cu, the plough layer was lower in the cropping systems compared with FW (difference 0.05 to 0.30 mg kg^-1^), whereas the plough sole was higher in GLR compared with other systems.

Ox-Cu was uniformly distributed in both soil layers across the cropping systems. The values generally followed the order: FW > GLR > WC > AC in the plough layer; the order changed to: GLR > WC > FW > AC in the plough sole. Om-Cu in the plough layer of GLR, AC, and WC relative to FW decreased by 25.81%, 19.35%, and 12.90%, respectively. Owing to the shallow roots, Om-Cu in the plough sole was 20% higher in WC compared with FW.

Min-Cu was higher in FW than in the cropping systems, and the values in both soil layers followed the order: FW > GLR > AC > WC. There was a greater reduction in Min-Cu in the plough sole than in the plough layer of cropping systems.

### Cu fractions under fertilisation practices

**Fig 3:**
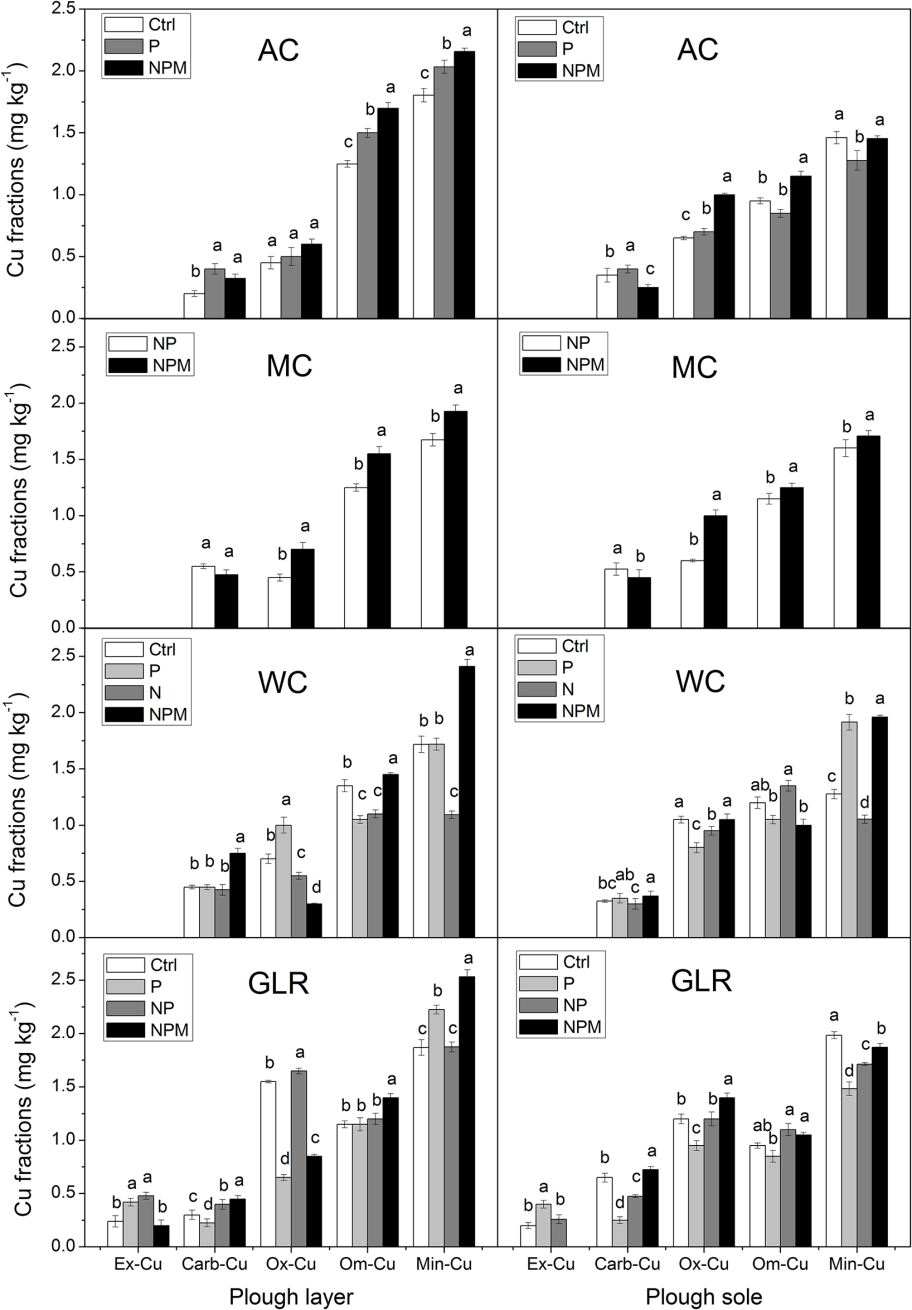
Soil Cu fractions in cropping systems with fertilisation treatments. The unit for Min-Cu is 10 mg·kg^-1^. Different letters indicate a significant difference at *p*<0.05; Error bars represent standard errors of the mean (*n = 3*). Cropping systems: FW, fallow control; AC, continuous alfalfa; MC, continuous maize; WC, continuous winter wheat; GLR, grain-legume rotation. Fertiliser treatments: Ctrl, unfertilised control; N, nitrogen; P, phosphorus; NP, N + P; NPM, N + P + manure. Cu fractions: Ex-Cu, exchangeable Cu; Carb-Cu, carbonate-bound Cu; Ox-Cu, oxide-bound Cu; Om-Cu, organic-matter-bound Cu; and Min-Cu, mineral-bound Cu.

Long-term fertilisation affected the concentrations of Cu in different soil fractions to varying degrees (Fig 3). Application of NPM fertiliser led to the lowest Ex-Cu in GLR relative to the other fertilised treatments, whereas P fertilisation increased Ex-Cu in GLR, regardless of soil depth. Ex-Cu showed an increase in both soil layers in NP treatment and the increase were diminished with soil depth.

Carb-Cu concentration was higher in the plough layer of NPM treatment relative to unfertilised treatment for AC, WC and GLR systems (Fig 3). NPM fertiliser had different effects on Carb-Cu in the plough sole, with the lowest value in AC and the highest values in WC and GLR. Additionally, the Carb-Cu concentration of P treatment increased in AC and decreased in GLR relative to the other fertilised treatments.

The Ox-Cu concentration of P treatment had a similar trend in both soil layers of AC and GLR systems (Fig 3). The impacts of NPM fertiliser on Ox-Cu varied with soil depth. Specifically, the values of WC and GLR systems were 57.1% and 45.2% lower in the plough layer compared with unfertilised treatment, but were highest in the plough sole among fertilised treatments.

Compared with unfertilised treatment, the Om-Cu concentration of P treatment was 36% higher in the plough layer in AC, while the values in the plough sole had no differences for all the cropping systems (Fig 3). Moreover, NPM fertilisation resulted in higher Om-Cu in both soil layers of AC and GLR systems.

The trend of Min-Cu concentration was consistent in both soil layers of P and NPM treatments. NPM fertilisation led to higher Min-Cu in the plough layer relative to the plough sole.

## Discussion

### Relationship between soil chemical properties and Cu bioavailability

Studies have reported the effect of soil chemical properties on Cu forms, which affect the concentration of DTPA-Cu in calcareous soil [15]. In order to verify the effect of chemical properties on Cu bioavailability in Heilu soil, we analysed the relationship between soil organic matter, available P, CaCO_3_, pH, and Cu bioavailability. Table 1 shows that DTPA-Cu was negatively correlated with soil organic matter (*r = –*0.161), available P (*r = –*0.380, *p<*0.01), and CaCO_3_ (*r = –*0.369, *p<*0.01). Owing to the narrow range of pH values (8.23–8.66), the positive correlation between pH and DTPA-Cu (*r =* 0.558, *p<*0.01) may not explain the relationship between soil pH and available Cu completely.

**Table 1:** Path and correlation coefficients of soil chemical properties to DTPA-Cu.

		Indirect path coefficients				
Fraction	Direct path coefficients	Organic matter	Available P	CaCO_3_	pH	*r* ^a^
**Organic matter**	0.157		-0.028	-0.103	-0.187	-0.161
**Available P**	-0.048	0.090		-0.130	-0.292	-0.380
**CaCO3**	-0.222	0.072	-0.028		-0.191	-0.369
**pH**	0.504	-0.058	0.028	0.084		0.558

^a^ *r*, correlation coefficient; *r*_0.05_ = 0.215, *r*_0.01_ = 0.280, *n* = 84.

The significant direct path coefficients of pH and CaCO_3_ indicated that these two factors directly affected the bioavailability of Cu in the Heilu soil. Soil components combined with metal ions are generally sensitive to pH changes [29]. At low pH, the high concentration of H^+^ suffered competitive adsorption with Cu^2+^ in soil solution, and decreased the adsorption capacity of soil clay minerals, thereby increasing the concentration of Cu^2+^; as the pH increased, the low concentration of H^+^ resulted in weak competitive adsorption with Cu^2+^ and high adsorption capacity of soil clay minerals, thereby decreasing the Cu bioavailability [5]. Cu appears to precipitate as carbonate hydroxides in calcareous soils [30]. Kalavrouziotis found that CaCO_3_ was negatively correlated with heavy metals and the increase of CaCO_3_ reduced the bioavailability of heavy metals in a soil cultivated with *Brassica oleracea* [31].

The nonsignificant direct path coefficient and significant indirect path coefficient of available P indicated that the factor indirectly affected the bioavailability of Cu through the change of pH in the Heilu soil. Although Cu^2+^ can form an insoluble salt with HPO_4_^2-^ [15], the influence of phosphate on Cu sorption was not due to the reaction between Cu^2+^ ions and sorbed phosphate. Rather, simulations suggested that the addition of phosphate raised pH levels, and hence the formation of readily sorbed Cu–P species [4]. The nonsignificant direct and indirect path coefficients of organic matter were indicative of the complex relationship between organic matter and DTPA-Cu, in agreement with the result of Fan et al. in all aggregates [32].

### Effect of cropping and fertilisation on available Cu

Compared to the status of available soil micronutrients in 1984, we observed decreased concentrations in all the treatments at the end of 2002. This may be due to the leaching losses of micronutrients in soil water as discussed by Gustafson et al. [33]. The concentrations of DTPA-Cu (0.52–1.02 mg kg^-1^) in the Heilu soil are higher than the critical level (0.5 mg kg^-1^) [16], indicating that available Cu is relatively rich in the study area. However, the average concentration of DTPA-Cu (0.71 mg kg^-1^) is less than the regional average of the Loess Plateau (0.93 mg kg^−1^) [16] and the lowest concentration of available Cu was close to the deficient concentration. Therefore, monitoring of Cu concentrations is needed to avoid its deficiency in soil.

Plant species show different responses to Cu [34]. Different external forms and internal structures of plants result in varying absorption capacity [35]. In the present study, long-term planting of crops led to a decrease in DTPA-Cu concentration and the decrease was greater in the plough layer with wheat than with legumes. The uptake of available Cu by crops decreased the DTPA-Cu concentration, while their varying absorption capacity caused the difference between treatments. Zhu et al. [36] found that micronutrients in leguminous crops were markedly lower than those in gramineous crops, indicating leguminous crops can enrich the soil in both macronutrients and micronutrients.

In the present study, fertilisation had no contribution to the bioavailability of Cu under long-term continuous cropping in the Heilu soil. This agrees with the result obtained by Li et al. [6] that DTPA-Cu did not change among inorganic or organic fertiliser treatments in an aquic inceptisol (winter wheat–summer maize rotation). However, Wang et al. [37] stated that the application of inorganic fertilizer only did not increase DTPA-Cu, whereas inorganic plus organic fertilisers increased DTPA-Cu in black soil (spring maize), red soil (winter wheat–summer maize rotation), and fluvo-aquic soil (winter wheat–summer maize rotation). The different results may be related to the variability of soil types, rotations, pH, and organic matter, among various factors.

### Effect of cropping and fertilisation on Cu fractions

Under different cropping patterns, soil water condition, pH, organic matter and redox potential are different, resulting in various changes in the micronutrient fractions [38, 39]. In the present research, high concentrations of Min-Cu and Om-Cu and low concentrations of Ox-Cu and Carb-Cu were obtained in the continuous cropping treatments. Similarly, the main Cu fraction in an albic soil was residual Cu followed by organic matter combined Cu, and the concentrations of Cu in other fractions were low after long-term continuous cropping of soybean [40].

However, the concentration of Ox-Cu in long-term rotation was high and followed Min-Cu only in the present study. Long-term studies have demonstrated the positive effects of crop rotation, especially with legumes, for maintaining soil organic matter content [41] and secreting organic acids and acidoids to remobilise Cu forms [42]. The organic acids released can form chelate with Cu ions, and the increase in organic acids will enhance their complexation ability. Then, the Cu ions adsorbed on the surface of soil particles will enter the soil solution to facilitate Cu uptake [43].

The presence of phosphate can favour and hinder the mobility of metal ions [44, 45]. Phosphate increases or decreases mobility depending on whether its effect on soil acidity is to decrease or increase pH [46]. The input of P fertiliser increased all the fractions of Cu under continuous cropping of alfalfa, while it decreased Carb-Cu, Ox-Cu and Om-Cu under grain–legumes rotation, in the plough layer. This indicated that cropping systems can result in different soil microenvironments [38, 39].

Organic matter has the ability to complex Cu, while Cu ion has a strong affinity to organic ligand [12]. The–OH and–COOH groups supplied by manure can increase the binding sites and combined with Cu to form insoluble, immobile complexes, thereby decreasing the concentration of free Cu^2+^ [47]. In the present study, the application of NPM increased Om-Cu and Min-Cu in the plough layer. Additionally, the application of organic fertiliser with NPK for 3 years increased Cu bound to organic matter and residual Cu in a paddy red soil [48].

The aqueous complexes of Cu and organic matter can be formed though not to the same extent as many synthetic chelating agents, and these complexes are not easily leached in the soil [49]. This was the case in the present study for Om-Cu, which occurred at higher levels in the plough layer than in the plough sole of the Heilu soil.

### Relationship of Cu fractions and indication for bioavailability

In order to reveal the relationship between Cu fractions and their bioavailability, we analysed the contribution of each Cu fraction to available Cu (DTPA-Cu) by correlation analysis and path analysis (Table 2). The results showed that Carb-Cu was negatively correlated with DTPA-Cu (*r = –*0.219, *p<*0.05). Carb-Cu represents the associated or co-precipitated fraction of Cu that has been occluded into carbonates, and the presence of carbonate can decrease available Cu in calcareous soil through the precipitation or occlusion effect [50, 51]. The acid-extractable (water-soluble, exchangeable and carbonate-bound) and organic bound fractions of Cu in soil have been used to predict plant uptake [10, 14]. Carb-Cu had the significant direct path coefficient and negative correlation coefficient to DTPA-Cu, suggesting that this fraction is a direct source of available Cu in the Heilu soil.

**Table 2:** Path and correlation coefficients of Cu fractions to DTPA-Cu. Carb-Cu, carbonate-bound Cu; Ox-Cu, oxide-bound Cu; Om-Cu, organic-matter-bound Cu; and Min-Cu, mineral-bound Cu.

		Indirect path coefficients				
Fraction	Direct path coefficients	Carb-Cu	Ox-Cu	Om-Cu	Min-Cu	*r* ^a^
**Carb-Cu**	-0.220		-0.003	0.009	-0.005	-0.219
**Ox-Cu**	-0.034	-0.021		0.056	0.001	0.002
**Om-Cu**	-0.237	0.008	0.008		-0.006	-0.227
**Min-Cu**	-0.017	-0.059	0.001	-0.078		-0.153

^a^ *r*, correlation coefficient; *r*_0.05_ = 0.215, *r*_0.01_ = 0.280, *n* = 84.

Ox-Cu was the only fraction positively correlated with DTPA-Cu (*r =* 0.002). Bradl and Cao et al. [52, 53] found that Cu in soil is first precipitated at stronger competitive sites as Fe and Mn oxides and then at weaker sites as carbonates. Fe and Mn oxides can make Cu ineffective to plants [54]. Except under reducing or low pH conditions, the stronger competitive adsorption means the metal oxides are difficult to be released [15]. The nonsignificant direct path coefficient and correlation coefficient of Ox-Cu indicated that this factor made negligible contribution to availability Cu in soil.

In the present study, Om-Cu was negatively correlated with DTPA-Cu, probably because the weak and specific fractions of Om-Cu had different effects on DTPA-Cu [15]. The largest significant direct path coefficient and correlation coefficient of Om-Cu indicated that this factor contributed the most to available Cu in the Heilu soil (Table 2), in agreement with the result of Shao and Zhen [55]. Since Cu has a stronger capacity to associate with the organic ligand [12], the contribution of Om-Cu to soil available Cu is mainly attributed to organic matter. Om-Cu can be used to predict plant uptake [10, 56] and this fraction is considered to be the major direct source of available Cu in the Heilu soil.

Min-Cu cannot be absorbed by plants and thus is not readily available to crops. The release and transformation of Min-Cu to other fractions are slow [57]. Therefore, Min-Cu made little direct contribution to Cu bioavailability in the Heilu soil, as indicated by its nonsignificant direct and indirect path coefficients (Table 2). Min-Cu was positively correlated with Carb-Cu(*r =* 0.267, *p<*0.05), and Om-Cu (*r =* 0.331, *p<*0.01), indicating that this fraction can reflect the potential supply capacity of Cu in soil.

**Fig 4:**
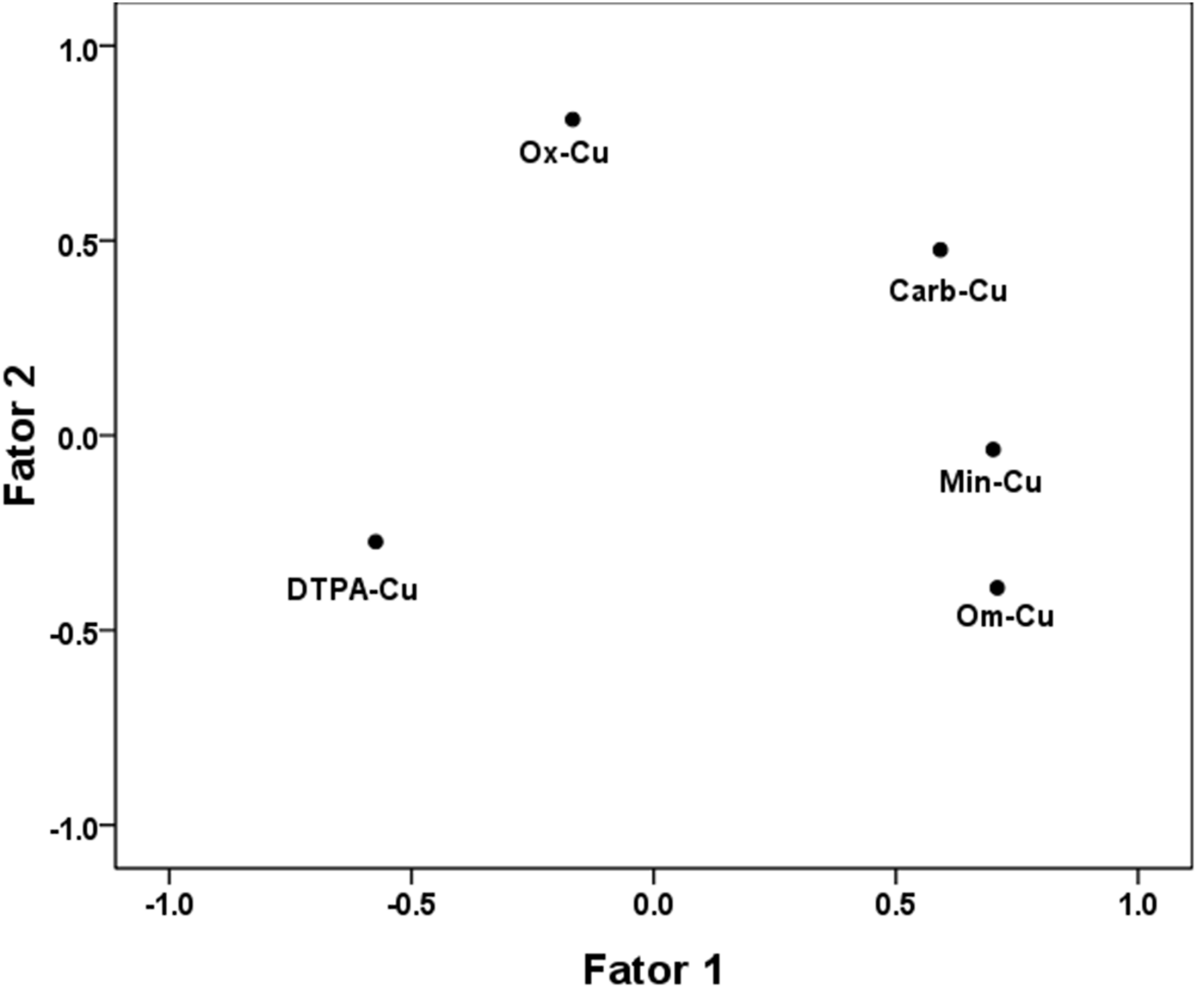
Factor plot of DTPA-Cu and Cu fractions. Carb-Cu, carbonate-bound Cu; Ox-Cu, oxide-bound Cu; Om-Cu, organic-matter-bound Cu; and Min-Cu, mineral-bound Cu.

Factor analysis was conducted to clarify the relationship between Cu fractions and DTPA-Cu. In the factor plot (Fig 4), Om-Cu and Carb-Cu were far away from DTPA-Cu. According the preceding analysis, Om-Cu and Carb-Cu were the major direct sources of available Cu. Their contributions to available Cu were crucial, as indicated by the significant correlation coefficients and direct path coefficients (Table 2). The high correlations between Om-Cu, Carb-Cu and Min-Cu resulted in the relative short distances, indicating that the three fractions can be classified as the sources of available Cu. Ox-Cu had little contribution to available Cu and its position was therefore farther away from DTPA-Cu.

In conclusion, this study elucidated the effects of long-term crop rotation and fertilisation on the bioavailability and distribution of Cu fractions in the Heilu soil. The bioavailability of Cu was greatly affected by cropping systems, and fertilisation had no effect on it in the continuous cropping treatments. Cu fractions changed with cropping systems, while NPM increased Om-Cu and Min-Cu in the plough layer. Om-Cu and Carb-Cu contributed directly to soil Cu bioavailability, and Min-Cu acted as an indicator of potential supply capacity of Cu in the soil. Although available Cu is relatively rich in the Heilu soil under long-term cropping and fertilisation, monitoring of Cu concentration is needed to avoid its deficiency in soil on the Loess Plateau.

## Supporting Information

S1 Table Soil DTPA-Cu 18 years after the start of the experiment (mg kg^-1^). (DOCX)

S2 Table Soil Cu fractions in different cropping systems (mg kg^-1^). (DOCX)

S3 Table Soil Cu fractions in cropping systems with fertilisation treatments (mg kg^-1^). (DOCX)

S4 Table Correlation of Cu fractions and DTPA-Cu. (DOCX)
