# Supplementary material for: Long-Term Effect of Crop Rotation and Fertilisation on Bioavailability and Fractionation of Copper in Soil on the Loess Plateau in Northwest China
Source: PLoS One. 2015 Dec 22;10(12):e0145370. doi: 10.1371/journal.pone.0145370 (PMC4687829; doi:10.1371/journal.pone.0145370)
Supplement: S1 Table — (DOCX) [file pone.0145370.s001.docx]

**Supporting Information**

**Long-term Effect of Crop Rotation and Fertilisation on Bioavailability and Fractionation of Copper in Soil on the Loess Plateau in Northwest China**

Yifei Zang^1^, Xiaorong Wei^2^, Mingde Hao^1,2^

*^1^ College of Natural Resources and Environment, Northwest A & F University, Yangling, Shaanxi, China*

*^2^ Institute of Soil and Water Conservation, Chinese Academy of Sciences and Ministry of Water Resources, Yangling, Shaanxi, China*

E-mail: zangyifei@126.com

**S1 Table Soil DTPA-Cu 18 years after the start of the experiment (mg kg^-1^)**

| **Layer** | **FW** | | **CC** | | | **MC** | | | | **WC** | | | | **CLR** | | | | |
| --- | --- | --- | --- | --- | --- | --- | --- | --- | --- | --- | --- | --- | --- | --- | --- | --- | --- | --- |
|  |  | **Ctrl** | | **P** | **NPM** | | **NP** | **NPM** | **Ctrl** | | **P** | **N** | **NPM** | | **Ctrl** | **P** | **NP** | **NPM** |
| Plough layer | 0.836 | 0.716 | | 0.734 | 0.703 | | 0.601 | 0.627 | 0.660 | | 0.631 | 0.612 | 0.686 | | 0.699 | 0.638 | 0.570 | 0.612 |
| Plough sole | 0.982 | 0.954 | | 0.858 | 0.890 | | 0.614 | 0.594 | 0.736 | | 0.703 | 0.698 | 0.662 | | 0.734 | 0.688 | 0.653 | 0.650 |
