# Supplementary material for: Long-Term Effect of Crop Rotation and Fertilisation on Bioavailability and Fractionation of Copper in Soil on the Loess Plateau in Northwest China
Source: PLoS One. 2015 Dec 22;10(12):e0145370. doi: 10.1371/journal.pone.0145370 (PMC4687829; doi:10.1371/journal.pone.0145370)
Supplement: S2 Table — (DOCX) [file pone.0145370.s002.docx]

**Supporting Information**

**Long-term Effect of Crop Rotation and Fertilisation on Bioavailability and Fractionation of Copper in Soil on the Loess Plateau in Northwest China**

Yifei Zang^1^, Xiaorong Wei^2^, Mingde Hao^1,2^

*^1^ College of Natural Resources and Environment, Northwest A & F University, Yangling, Shaanxi, China*

*^2^ Institute of Soil and Water Conservation, Chinese Academy of Sciences and Ministry of Water Resources, Yangling, Shaanxi, China*

E-mail: zangyifei@126.com

**S2 Table Soil Cu fractions in different cropping systems (mg kg^-1^)**

| Layer | System | Ex-Cu | Carb-Cu | Ox-Cu | Om-Cu | Min-Cu |
| --- | --- | --- | --- | --- | --- | --- |
| Plough layer | FW | -- | 0.500 | 1.600 | 1.550 | 19.794 |
|  | AC | -- | 0.200 | 0.450 | 1.250 | 18.049 |
|  | WC | -- | 0.450 | 0.700 | 1.350 | 17.179 |
|  | GLR | 0.240 | 0.300 | 1.550 | 1.150 | 18.696 |
| Plough sole | FW | -- | 0.325 | 0.900 | 1.000 | 21.616 |
|  | AC | -- | 0.350 | 0.650 | 0.950 | 14.616 |
|  | WC | -- | 0.325 | 1.050 | 1.200 | 12.766 |
|  | GLR | 0.200 | 0.650 | 1.200 | 0.950 | 19.848 |
