# Supplementary material for: Long-Term Effect of Crop Rotation and Fertilisation on Bioavailability and Fractionation of Copper in Soil on the Loess Plateau in Northwest China
Source: PLoS One. 2015 Dec 22;10(12):e0145370. doi: 10.1371/journal.pone.0145370 (PMC4687829; doi:10.1371/journal.pone.0145370)
Supplement: S3 Table — (DOCX) [file pone.0145370.s003.docx]

**Supporting Information**

**Long-term Effect of Crop Rotation and Fertilisation on Bioavailability and Fractionation of Copper in Soil on the Loess Plateau in Northwest China**

Yifei Zang^1^, Xiaorong Wei^2^, Mingde Hao^1,2^

*^1^ College of Natural Resources and Environment, Northwest A & F University, Yangling, Shaanxi, China*

*^2^ Institute of Soil and Water Conservation, Chinese Academy of Sciences and Ministry of Water Resources, Yangling, Shaanxi, China*

E-mail: zangyifei@126.com

**S3 Table Soil Cu fractions in cropping systems with fertilisation treatments (mg kg^-1^)**

| System | Layer | Fertilasition | Ex-Cu | Carb-Cu | Ox-Cu | Om-Cu | Min-Cu |
| --- | --- | --- | --- | --- | --- | --- | --- |
| AC | Plough layer | Ctrl | -- | 0.200 | 0.450 | 1.250 | 18.049 |
|  |  | P | -- | 0.400 | 0.500 | 1.500 | 20.338 |
|  |  | NPM | -- | 0.325 | 0.600 | 1.700 | 21.581 |
|  | Plough sole | Ctrl | -- | 0.350 | 0.650 | 0.950 | 14.616 |
|  |  | P | -- | 0.400 | 0.700 | 0.850 | 12.777 |
|  |  | NPM | -- | 0.250 | 1.000 | 1.150 | 14.547 |
| MC | Plough layer | NP | -- | 0.550 | 0.450 | 1.250 | 16.748 |
|  |  | NPM | -- | 0.475 | 0.700 | 1.550 | 19.267 |
|  | Plough sole | NP | -- | 0.525 | 0.600 | 1.150 | 16.014 |
|  |  | NPM | -- | 0.450 | 1.000 | 1.250 | 17.086 |
| WC | Plough layer | Ctrl | -- | 0.450 | 0.700 | 1.350 | 17.179 |
|  |  | P | -- | 0.450 | 1.000 | 1.050 | 17.198 |
|  |  | N | -- | 0.425 | 0.550 | 1.100 | 10.921 |
|  |  | NPM | -- | 0.750 | 0.300 | 1.450 | 24.106 |
|  | Plough sole | Ctrl | -- | 0.325 | 1.050 | 1.200 | 12.766 |
|  |  | P | -- | 0.350 | 0.800 | 1.050 | 19.157 |
|  |  | N | -- | 0.300 | 0.950 | 1.350 | 10.535 |
|  |  | NPM | -- | 0.370 | 1.050 | 1.000 | 19.617 |
| GLR | Plough layer | Ctrl | 0.240 | 0.300 | 1.550 | 1.150 | 18.696 |
|  |  | P | 0.420 | 0.225 | 0.650 | 1.150 | 22.261 |
|  |  | NP | 0.480 | 0.400 | 1.650 | 1.200 | 18.747 |
|  |  | NPM | 0.200 | 0.450 | 0.850 | 1.400 | 25.330 |
|  | Plough sole | Ctrl | 0.200 | 0.650 | 1.200 | 0.950 | 19.848 |
|  |  | P | 0.400 | 0.250 | 0.950 | 0.850 | 14.838 |
|  |  | NP | 0.260 | 0.475 | 1.200 | 1.100 | 17.135 |
|  |  | NPM | -- | 0.725 | 1.400 | 1.050 | 18.708 |
