# Supplementary material for: Long-Term Effect of Crop Rotation and Fertilisation on Bioavailability and Fractionation of Copper in Soil on the Loess Plateau in Northwest China
Source: PLoS One. 2015 Dec 22;10(12):e0145370. doi: 10.1371/journal.pone.0145370 (PMC4687829; doi:10.1371/journal.pone.0145370)
Supplement: S4 Table — (DOCX) [file pone.0145370.s004.docx]

**Supporting Information**

**Long-term Effect of Crop Rotation and Fertilisation on Bioavailability and Fractionation of Copper in Soil on the Loess Plateau in Northwest China**

Yifei Zang^1^, Xiaorong Wei^2^, Mingde Hao^1,2^

*^1^ College of Natural Resources and Environment, Northwest A & F University, Yangling, Shaanxi, China*

*^2^ Institute of Soil and Water Conservation, Chinese Academy of Sciences and Ministry of Water Resources, Yangling, Shaanxi, China*

E-mail: zangyifei@126.com

**S4 Table Correlation of Cu fractions and DTPA-Cu**

|  | Carb-Cu | Ox-Cu | Om-Cu | Min-Cu | DTPA-Cu |
| --- | --- | --- | --- | --- | --- |
| Carb-Cu | 1.000 | 0.096 | -0.037 | 0.267 | -0.219 |
| Ox-Cu |  | 1.000 | -0.237 | -0.043 | 0.002 |
| Om-Cu |  |  | 1.000 | 0.331 | -0.227 |
| Min-Cu |  |  |  | 1.000 | -0.153 |
| DTPA-Cu |  |  |  |  | 1.000 |
